# Revisiting the Antigen-Presenting Function of β Cells in T1D Pathogenesis

**DOI:** 10.3389/fimmu.2021.690783

**Published:** 2021-07-14

**Authors:** Yang Li, Fei Sun, Tian-Tian Yue, Fa-Xi Wang, Chun-Liang Yang, Jia-Hui Luo, Shan-Jie Rong, Fei Xiong, Shu Zhang, Cong-Yi Wang

**Affiliations:** The Center for Biomedical Research, Department of Respiratory and Critical Care Medicine, NHC Key Laboratory of Pulmonary Diseases, Tongji Hospital, Tongji Medical College, Huazhong University of Science and Technology, Wuhan, China

**Keywords:** β cell, antigen presentation, autoimmune diabetes, innate immunity, crosstalk

## Abstract

Type 1 diabetes (T1D) is characterized by the unresolved autoimmune inflammation and islet β cell destruction. The islet resident antigen-presenting cells (APCs) including dendritic cells and macrophages uptake and process the β cell-derived antigens to prime the autoreactive diabetogenic T cells. Upon activation, those autoreactive T cells produce copious amount of IFN-γ, TNF-α and IL-1β to induce β cell stress and death. Autoimmune attack and β cell damage intertwine together to push forward this self-destructive program, leading to T1D onset. However, β cells are far beyond a passive participant during the course of T1D development. Herein in this review, we summarized how β cells are actively involved in the initiation of autoimmune responses in T1D setting. Specifically, β cells produce modified neoantigens under stressed condition, which is coupled with upregulated expression of MHC I/II and co-stimulatory molecules as well as other immune modules, that are essential properties normally exhibited by the professional APCs. At the cellular level, this subset of APC-like β cells dynamically interacts with plasmacytoid dendritic cells (pDCs) and manifests potency to activate autoreactive CD4 and CD8 T cells, by which β cells initiate early autoimmune responses predisposing to T1D development. Overall, the antigen-presenting function of β cells helps to explain the tissue specificity of T1D and highlights the active roles of structural cells played in the pathogenesis of various immune related disorders.

## Introduction

Since the early 1970s, type 1 diabetes (T1D) has been defined as an autoimmune disorder resulting from the intolerance to pancreatic β cell derived auto-antigens (1, 2), and subsequent studies have consistently demonstrated that islet β cell dysfunction and immune cell autoreactive response contribute to disease progression, while the exact mechanisms largely remain unknown. In the canonical paradigm, the damaged β cells expose self-antigens to resident or patrolling antigen presenting cells (APC) to initiate the immune process (3, 4). Indeed, polymorphisms within the class II major histocompatibility complex (MHC II) (e.g., I-A^g7^ in non-obese diabetic (NOD) mice and HLA-DQ8 in human counterparts), strongly correlate with T1D propensity. Such MHC haplotype generates the anchoring site of MHC molecules that favors the binding of self-peptide, thereby facilitating the subsequent APC-T cell interaction (4). Other genetic predisposing factors further exacerbate the immune reactivity against β cells (5, 6). Given many of the identified autoantigens are not confined to islet and the autoreactive T cells constantly patrol in the peripheral circulation, it is, therefore, hard to explain the tissue specificity of T1D.

Accumulating evidence reveals that structural cells are not mere passive participants in immune related disorders. They can actively produce cytokines, chemokines, and even the MHC II molecules, a hallmark that is traditionally considered as a privilege of professional APCs. The emerging concept of “structural cell immunology” blurs the boundary between immune cells and non-immune cells, extends our understanding of the immune response initiation, and provides a chance to re-scrutinize the established disease pathogenesis (7). This concept is extensively corroborated in the tumor microenvironment (TME), where non-hematopoietic cells such as tumor cells, fibroblasts and other mesenchymal cells dynamically shape the anti-tumor immune response. This is also the case in other immune engaged disorders. For example, obese mice have an obvious increase in average adipocyte size, and the larger adipocytes express higher level of MHC II than the smaller ones, which directly activate T cells during the course of obesity (8–10). Endothelial cells (ECs) express Toll-like receptor 4 (TLR4) and receptors for TNF-α and IL-1β, and therefore, LPS stimulation activates ECs to produce pro-inflammatory cytokines and chemokines, which then recruit immune cells and propagate the immune response (10). Notably, under certain conditions, ECs express both MHC I and MHC II and directly present antigens to T cells by acting as APC like cells (11). In rheumatoid arthritis (RA), the fibroblast-like synoviocytes (FLS) in the inflamed synovium share similar intrinsic properties as the follicular dendritic cells (FDCs), which help to attenuate apoptosis of germinal center (GC) B lymphocytes and exacerbate disease progression (12). These previously unappreciated immune functions of non-immune cells give us insights that abnormality of structural and/or stromal cells may well interpret the etiological origin of autoimmune diseases.

It was noted that cytokines such as IFN-γ, TNF-α and IL-1β following autoimmune attacks elicit ER and oxidative stress to cause β cell damage. The impaired β cells, however, are able to secret chemokines to motivate more immune cells and the damage associated molecular patterns (DAMPs) to push forward this vicious cycle of crosstalk (13). Nonetheless, two critical questions are yet to be elucidated: what happened to β cells even before an obvious autoimmune strike? Would β cells be both victims and culprits in early T1D pathogenesis? In fact, β cells have the ability to express MHC and costimulatory molecules to prime the adaptive immune response. Based on such intriguing findings, we herein intend to summarize the characteristics of APC-like β cells and to sort out factors that endow β cell with the antigen-presenting function. We would also discuss how APC-like β cells regulate T1D development and highlight the potential intervention strategies against T1D in clinical settings.

## Presence of APC-Like β Cells in the Islets

Antigenic peptide MHC complex (pMHC) provides the first signal for antigen presentation. Most islet antigens have been identified by HLA binding/tetramers, including insulin, proinsulin, islet antigen 2 (IA-2) and glutamic acid decarboxylase 65 (GAD65) (14–17). Other antigens such as islet amyloid polypeptide (IAPP) and glucose-regulated protein 78 (GRP78) have been distinguished through analysis of antigen-specific T cells (18). MHC class I, expressed essentially in all nucleated cells, presents intracellular peptides onto β cell surface, which directly leads to the activation of CD8^+^ T cells, an early feature of T1D development (19). CD4 T cells which specifically recognize peptide MHC class II complex, exert effector function and help B cells in autoantibody generation, which is essential to drive prolonged islet inflammation (20). Initially, most studies have considered that MHC class II is exclusively expressed in local professional APCs such as dendritic cells and macrophages (21). Intriguingly, several studies suggested that a proportion of β cells from type 1 diabetic patients or NOD mice also express MHC class II molecules (22–24). Similarly, RNA-Seq and immunohistological analysis demonstrated that β cells from recent-onset type 1 diabetic donors express MHC class II and its transcriptional regulator class II major histocompatibility complex trans-activator (CIITA) protein, which was hardly detectable in the islet cells of non-diabetic donors (25). Moreover, the I-Ag^7^ expressed β cells isolated from islets of diabetic NOD mice could independently induce proliferation of CD4^+^ T cells *in vitro* (26). In this case, β cells may serve as the APC-like cells in presenting autoantigen to activate islet-infiltrating CD4^+^ T cells.

Other than acquisition of MHC molecule, a set of second signals are also required to act as APCs. For instance, co-stimulatory molecules and cell-adhesion molecules, key components in the formation of immunological synapse, are necessary for the optimal activation of antigen-specific T cells. Although no evidence shows the expression of B7-1/B7-2 (CD80/CD86) on human pancreatic β cells, transgenic overexpression of B7-1 on NOD pancreatic islet accelerates the progression of type 1 diabetes (27, 28). Clustering of T cells with APCs is primarily mediated by the interaction between lymphocyte function-associated-1 (LFA-1) on the surface of lymphocytes and the intercellular adhesion molecule-1 (ICAM-1) on the APC cells. ICAM-1 was not expressed on the surface of normal human islet cells, but it can be detected following a 72h induction by IFN-γ or TNF-α (29). Collectively, these lines of evidence indicate that islet β cells display essential phenotypic characteristics that are normally possessed by the classical APCs, which supports the idea that β cells actively engage in the initiation of autoimmune response and is responsible for their own demise in T1D pathogenesis.

## Potential Triggers for the Formation of APC-Like β Cells

In β cell, generation of neoepitopes is associated with initial loss of immune tolerance, evidenced by local infiltration of effector T cells and the emergence of autoantibodies. Post-translational modification (PTM) affects protein properties, which is generally involved in normal physiological process including establishment of immune tolerance during the thymic and peripheral selection (30). In some cases, however, abnormal PTM process might alter protein structure to create novel β cell-specific epitopes that are not tolerated by the immune system. For example, citrulline modified GAD65 in β cells elicits T cell response in T1D patients (15), and similar modification of GRP78 was observed in the pancreatic islet of NOD mice (31). This kind of changes in PTM can be induced by reactive oxygen species (32) and inflammatory cytokines, suggesting the relevance between β cellular stress and neoepitope formation (33, 34). Endoplasmic reticulum (ER) is necessary for β cell functionality since it undertakes biosynthesis of proinsulin. Under physiological condition, up to 20% of proinsulin fails to achieve the valid conformation, so the ER associated degradation (ERAD) and the unfolded protein response (UPR) signaling are critical for maintaining ER homeostasis. However, upon exposure to stressful microenvironments such as proinflammatory cytokines and ROS, proinsulin is more susceptible to undergo misfolding, which induces over-active UPR to cause ER stress (35). This pathological process could result in abnormal PTM of other proteins or direct generation of neoepitopes, which are then processed and presented by β cells and/or APCs (36). The analysis of NOD islet revealed that β cells deficient in IRE1α, a key component of ER stress, declines the expression of autoantigen and MHC class I complex (37). The hybrid insulin peptides (HIPs), another type of autoantigen in T1D, have been identified by sequencing epitopes from β cells in diabetic NOD mice. The fused peptide is produced through combination of proinsulin peptides with other peptides in β cell secretory granules, leading to a chimeric antigen with enhanced MHC binding affinity and the ability to break the immune tolerance (38). Unfortunately, the understanding of HIP formation remains at the phenomenal levels thus far, the underlying mechanisms are yet to be elucidated. Additional processes such as alternative RNA splicing also contributes to generation of neoepitopes. For example, mRNA splice peptide SCG5009, the product generated from the *Secretogranin V* gene, was processed by HLA molecules and presented to T cells in the pancreas to initiate T1D (39). In line with those observations, DNA and protein methylation events elicited by inflammatory cytokines or other stress signals have been found related to abnormal β cell activity and insulin expression (40).

Although the detailed mechanism largely remains elusive, the generation of neoantigens is apparently a consequence of β cell stress. The stress response can either stem from intrinsic cell abnormalities or external cytokine stimulations, which induce or exaggerate β cell dysfunction. The interferon (IFN) family contains IFN-α/β (type I) and IFN-γ (type II), both of which are strongly associated with T1D pathogenesis (41–43). IFN-α is a critical cytokine produced by the immune system against foreign virus or tissue damage (43, 44), while IFN-γ is secreted by T cells or NK cells with high potency in propagating islet inflammation (45). It is known that pancreatic IFN-α upregulates the expression of MHC I and costimulatory molecules on the pancreatic β cells in T1D patients, leading to autoantigen presentation and activation of cytotoxic CD8^+^ T cells, which is considered as the early event of T1D (46, 47). Importantly, IFN-α alone, or IFN-γ plus TNF-α could induce MHC II gene expression in human islet coupled with the expression of the CIITA isoform (48, 49). Therefore, the IFN family members possess the ability to up-regulate the expression of MHC and molecules relevant to antigen presentation on the surface of islet β cells. As a result, external inflammatory cytokine stimulation and/or intrinsic β cell dysfunction induce neoantigen generation along with the expression of MHC and co-stimulatory molecules, which eventually endow β cells with antigen-presenting property (**Table 1**).

**Table 1:** Key features of APC-like islet β cells.

Key features	Induced by	Effect	Reference
**Neo-autoantigen generation**			
Post-translationally modified peptides	ROS, ER stress	Abnormal modification of GAD65, GRP78 etc.; generation of neoepitopes	(15, 31, 33–35)
Hybrid insulin peptides	Islet inflammation	Producing chimeric antigen with enhanced MHC binding affinity; breaking immune tolerance	(38)
RNA splicing products	Inflammatory cytokines	Generation of non-self epitopes; activation of pancreas-infiltrating CD8^+^ T lymphocytes	(39)
**MHC molecule expression**			
MHC-I	IFN-α	Presentation of intracellular peptides and activation of CD8^+^ T cells	(19)
MHC-II	IFN-α; IFN-γ plus TNF-α	Activation of islet-infiltrating CD4^+^ T cells	(22–24)
**Co-stimulatory molecule expression**			
B7-1	–	Providing the co-stimulatory signal for T cell activation	(27)
ICAM-1	IFN-γ, TNF-α	Facilitating the interaction between antigen presenting cell and T cell	(29)

## Dynamic Crosstalk Between APC-Like β Cells and Immune Cells

It is believed that viral infection (Coxsackievirus in particular) is related to T1D pathogenesis, at least in part, through stimulating the production of type I IFN both in β cells and plasmacytoid dendritic cells (pDCs). Compared to β cells, pDCs have the ability to produce large amounts of IFN-α and IFN-β, which in turn act on β cells to induce the APC-like phenotype (50). IFN-α producing pDCs have been detected in the blood of T1D patients at their first diagnosis (51). In consistent with this observation, IFN-α therapy in patients with hepatitis C virus infection or leukemia is associated with an increased risk for developing T1D (32, 52). However, some studies also revealed that pDCs exhibit a negative regulatory role in T1D setting, as NOD mice without pDCs manifest exacerbated insulitis (53). Such contradiction implies a dual role for pDCs in T1D pathogenesis, which depends on the microenvironment created by β cells and other cell types. Indeed, pDCs present antigens acquired exogenously in a tolerogenic manner, thereby attenuating CD8^+^ T cell proliferation (54). However, upon the presence of β cell damage, innate immune cells such as B-1a cells produce double-stranded DNA specific IgGs, and neutrophils generate DNA-binding cathelicidin-related antimicrobial peptide (CRAMP), both of which can activate pDCs to secrete IFN-α in the pancreatic islets (55). Therefore, pDCs not only sense the exogenous viral infection for immune defense, but also alarm the endogenous tissue damage signal to transform β cells into APC-like cells.

Accumulated evidence indicates that both CD4^+^ and CD8^+^ T cells are implicated in β cell destruction (56–58). Islet reactive T cells are initially primed and activated in the draining lymph nodes, after which they get entrance into the islet *via* pancreatic vasculature, leading to massive damage of β cells (59, 60). CD8^+^ T cell is a heterogenous population consisting of CD8^+^ CD28^-^ T suppressor subset, which exhibits defect in pathologic immune responses (61, 62), and the cytotoxic subset, which is paramount in inducing β cell death. Insulin peptides are the typical autoantigens for type 1 diabetic patients. It was found that peptide derived from insulin B chain is processed by the proteasomes, and then translocated into the endoplasmic reticulum *via* the peptide transporter TAP1, by which they bind to HLA-A2 onto β cell surface to serve as the major target for cytotoxic CD8^+^ T cell recognition (63). A recent study suggested that the circulating islet-reactive CD8^+^ T cells are predominantly naïve and largely overlapped between T1D and healthy subjects (39), in which HLA I peptidomics and transcriptomic analysis were combined to identify the epitopes presented by β cells in T1D patients and healthy donors. It was interestingly noted that antigens processed by β cells through multiple pathways for HLA-A2 restricted presentation is crucial to activate circulating CD8^+^ T cells (39).

Although the MHC II expressed β cells are capable of activating CD4^+^ T cells, the detailed mechanisms, however, are not yet to be elucidated. Autophagy is an intracellular system that delivers damaged organelles and cytosolic proteins to lysosomes for degradation (64). Autophagy dependent processes participate in restricted antigen presentation through lysosome contained proteases, and promote MHC II presentation of peptides from intracellular source (65). For instance, studies found that CD4^+^ T cells could specifically recognize citrullinated self-peptide presented by APCs, and autophagy plays a central role in the presentation of the post-translationally modified intercellular protein (66). It is therefore possible that β cells achieve the MHC II restricted cross-presentation of intracellular self-antigen in a manner similar as autophagy, which demands further investigations.

B cells are also central to T1D development. According to the functional specification, B cells are classified into three major subpopulations: the innate-like B1 cells (with CD5^high^ for B1a and CD5^low^ for B1b), the adaptive B2 cells (commonly noted B cells) and the regulatory B cell subset (Breg, marked by IL-10 production). As mentioned above, B1a cells are involved in T1D initiation by producing anti-dsDNA antibody upon sensing the β cell debris. However, this process is antigen non-specific and the antigen specific responses mediated by B2 cells may play an even greater role. Despite the presence of Breg subset, B cells are predominantly harmful as evidenced by the beneficial effect of total B cell depletion agents (e.g., anti-CD20 and anti-BAFF) (67, 68). B cells promote T1D progression by producing autoantibodies at the early phase and by presenting antigen to diabetogenic CD4^+^ and CD8^+^ T cells at the later phase. It is intriguing to note that the insulin reactive B cells are present in both T1D-prone NOD mice and T1D-resistant C57BL/6 mice (69). How the anergic state of B cells being breached in NOD mice and T1D patients is a big scientific question to answer. Cambier et al. revealed that BCR affinity, permissive pancreatic niche and abnormality in tolerance-regulating genes are essential for B cell mediated T1D pathogenesis (70). Moreover, the apparent transience of anergic B cell loss may well suggest that the loss of B cell anergy is a consequence of environmental insults, such as infection, injury and/or diet change (71). Thus, the APC-like β cells could aid in B cell escape of tolerance, resulting in an overt autoreactive B cell response.

## Summary and Perspective

Traditionally, professional APCs are considered to be the spotlight in T1D initiation. A population of CD11c^+^ CD103^+^ DCs relying on a transcriptional factor, the basic leucine zipper transcription factor AFT-like 3 (BATF3), has been identified in the murine islet. Those DCs are thought responsible for taking up and presenting antigens derived from secretory β cell granules and exogenous denatured proteins (72). Their presence in NOD mice is around 3- to 4-week of age, by then CD4^+^ T cells simultaneously enter into the islet. CD103^+^ DCs are also capable of cross-presenting and loading extracellularly acquired antigens onto MHC I to activate the autoreactive CD8^+^ T cells (73, 74). Another important APC population is macrophage. Islet macrophages, originating from precursors from yolk sac, fetal liver and bone marrow, reside in the pancreas since embryonic development (75, 76). Lower phagocytotic activity of NOD macrophages compared to that of BALB/c (a mouse strain that is not prone to T1D) is suggested to impede the defective clearance of cell debris generated during the physiological β mass turnover, which then predisposes to T1D initiation (77). Studies in 3-week old NOD, NOD.Rag1^-/-^ and B6.g7 mice revealed that islet macrophages are in an activated state with highly expressed MHC II, TNF and IL-1β, which are comparable to macrophages in the barrier surface such as lung and intestinal tract (78). Single cell analysis of gene expression profile indicated that macrophages in NOD islet are more inflamed as manifested by the upregulated interferon signature genes including *Cxcl2*, *Cxcl9* and *Ccl5* (78). Therefore, depletion of islet macrophages with CSF-1 receptor monoclonal antibody impairs the presentation of insulin epitopes from destroyed islet cells and delays the entrance of DCs and CD4^+^ T cells, thereby preventing T1D development in NOD mice (76).

**Figure 1:**
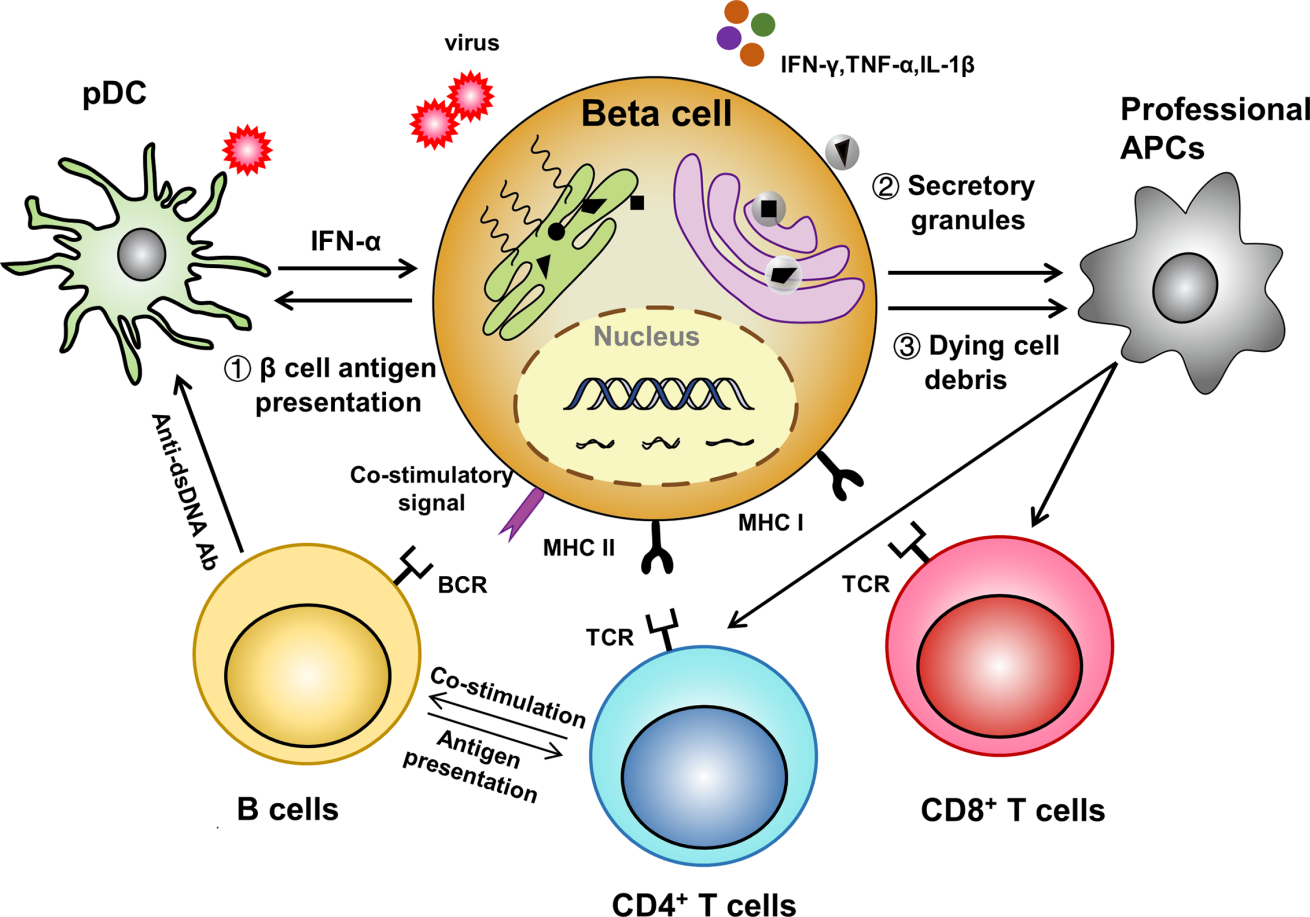
A β cell centered view of antigen-presentation. (1) Stressed APC-like β cells directly present antigens to activate adaptive immune cells; (2) Dysfunctional β cells secret autoantigen containing granules which are subsequently up-taken and processed by professional APCs; (3) Dying β cells themselves and the released pro-inflammatory molecules further amplify the antigen presenting process.

Undoubtedly, the pancreatic islet niche is crucial for T1D development. For instance, vascular endothelial growth factor (VEGF), which was found highly enriched in the serum of systemic lupus erythematosus patients (79), is also abundant in T1D blood. However, circulating plasma levels of VEGF do not correlate to metabolic control in long-standing T1D and the levels are not affected by the presence of microvascular complications (80). In contrast, locally increased VEGF promotes islet vascular remodeling and facilitates lymphocytic infiltration (81). APC-like β cell is another intriguing concept that extends our understanding of the etiology underlying organ specific autoimmunity. Given the fact that autoreactive T cells are indeed also present in healthy individuals (82), antigen presentation that lowers the threshold for aberrant T cell activation thus becomes critical in the initiation of autoimmune diseases including T1D. First, stressed β cells themselves actively present antigens to effector T cells. Second, dysfunctional β cells secret granules containing modified neoantigens and/or denatured proteins to be taken up by the adjacent professional APCs (83, 84). Third, the dying β cells release large amounts of autoantigens together with DAMPs (e.g., HMGB1 as an alarmin) to substantially activate the surrounding professional APCs. The general idea is that T1D is an autoimmune disorder which both begins and ends up with β cell death. Physiological β cell turnover is transient and self-controlled, while pathological β cell death leads to unrestrained inflammatory response probably due to the defective clearance by phagocytes and the occurrence of secondary necrosis (85). Furthermore, many T1D susceptible genes are expressed in β cells and make them vulnerable to death upon insults such as viral infection and inflammatory cytokine stimulation (86). Taken as a whole, this may reflect that the antigen presentation process in T1D setting is more diverse and intricate than what we previously thought, and the microenvironment shaped by structural cells, immune cells and their extensive cross-talk is pivotal for T1D development (**Figure 1**).

It is noteworthy that the ectopic expression of immune modules in β cells is not confined to the antigen-presenting function. For instance, it has been discovered that inflammatory cytokines stimulate human β cells to express negative-regulatory costimulatory molecules, B7-H4 and PD-L1, which could then serve as a counterbalance for derailed T cell response (87, 88). Moreover, islet cells are able to produce chemokines and cytokines such as CCL2, CCL22, IL-6 and so on to exhibit either pro-inflammatory or anti-inflammatory effect (89–91). Another good example is the TLR family proteins, in which TLR3/9 are related to the anti-viral response, while TLR2/4 activation induces MyD88 dependent transcription of inflammatory mediators and Erk dependent cell cycle arrest of β cells (92, 93). Particularly, current immunotherapies were predominantly designed to suppress the functionality of autoreactive immune cells along with tolerance induction, thereby holding back the overdriven immune responses. In particular, vaccination strategies based on β cell derived autoantigens have shown therapeutic efficacy by inducing antigen specific immune tolerance. This could be achieved through oral, intranasal or parenteral (subcutaneous and intramuscular) administration of each single autoantigen such as insulin or GAD65 (94, 95). Alternatively, multiple antigenic epitopes could be integrated into one polypeptide by means of the plasmid DNA platform (96, 97). The discovery of β cells to act as a part-time APC provides novel insights that manipulation of the expression pattern of immune modules in β cells holds the potential in early T1D treatment and prevention of organ rejection following transplantation of genetically engineered, immune-evasive islets.

In summary, herein we provided a β cell centered view in T1D pathogenesis (**Figure 1**). Evidence derived from current studies suggested an important role of structural cells in the initiation of T1D development, which could also be the case in other autoimmune disorders. However, additional studies would be necessary to translate those discoveries into clinical settings for prevention and treatment of type 1 diabetes.

## Author Contributions

YL, FS, and T-TY wrote the manuscript. F-XW, C-LY, J-HL, S-JR, and FX gave us valuable suggestions and made critical revisions. SZ and C-YW conceptualized and supervised the preparation of this manuscript. All authors contributed to the article and approved the submitted version.

## Funding

Our study was supported by the National Natural Science Foundation of China (81920108009, 81530024, 91749207, 81770823 and 81670729), the Ministry of Science and Technology (2016YFC1305002 and 2017YFC1309603), NHC Drug Discovery Program (2017ZX09304022-07), the Department of Science and Technology of Hubei State (2017ACA096), the Integrated Innovative Team for Major Human Disease Programs of Tongji Medical College, Huazhong University of Science and Technology, and the Innovative Funding for Translational Research from Tongji Hospital.

## Conflict of Interest

The authors declare that the research was conducted in the absence of any commercial or financial relationships that could be construed as a potential conflict of interest.
